# Syndromic surveillance of female sexually transmitted infections in primary care: a descriptive study in Monastir, Tunisia, 2007─2017

**DOI:** 10.1186/s12889-021-11647-2

**Published:** 2021-09-06

**Authors:** Wafa Dhouib, Imen Zemni, Meriem Kacem, Cyrine Bennasrallah, Manel Ben Fredj, Hela Abroug, Samia Grira, Maha Mastouri, Asma Sriha Belguith

**Affiliations:** 1 Department of Epidemiology and Preventive Medicine, University of Monastir, Monastir, Tunisia; 2 The Regional Direction of Primary Health of Monastir, Monastir, Tunisia; 3 Microbiology Laboratory, Monastir, Tunisia

**Keywords:** Sexually transmitted diseases, Vaginal discharge, *C. trachomatis*, *Neisseria gonorrhoeae*, Syphilis, Trichomonas vaginalis, *Gardnerella vaginalis*

## Abstract

**Background:**

Sexually Transmitted Infections (STIs) are a public health problem, especially for reproductive-age women. The aim of this study was to determine the incidence and trend of STIs during 11 years in Tunisia (2007–17).

**Methods:**

We conducted a descriptive study including all women with curable STIs (*chlamydia, gonorrhea*, syphilis and *trichomoniasis)* diagnosed with the syndromic approach in all basic health care centers of the Governorate of Monastir (Tunisia) from 2007 to 2017. Syndromes included, Pelvic Pain (PP), Vaginal Discharge (VD) and Genital Ulceration (GU).

**Results:**

We analyzed 40,388 episodes of curable STIs with a crude incidence rate and age standardized incidence rate of 1393 (95% Confidence Interval (CI); 1348–1438) / 100,000 Person Year (PY) and 1328 (95%CI; 1284–1372) /100,000 PY respectively. The incidence rate showed a positive trend over 11 years for all age groups and syndromes. VD was the most common syndrome with a crude incidence rate of 1170/100,000 PY. For all syndromes, women aged 20 to 39 were the most affected age group (*p* < 0.001).

**Conclusion:**

In conclusion, the incidence rate of STIs episodes among women diagnosed with the syndromic approach was high, consistent with the global evidence. Focusing on reviewing STIs surveillance system in low and middle-income countries could allow the achievement of the ending of STIs epidemics by 2030.

**Supplementary Information:**

The online version contains supplementary material available at 10.1186/s12889-021-11647-2.

## Background

Around the world, Sexually Transmitted Infections (STIs) are a public health problem being one of the leading causes of morbidity and mortality. World Health Organization’s (WHO) 2012 and 2016 estimates of the global prevalence and incidence of curable STIs (*chlamydia, gonorrhoea, trichomoniasis,* and *syphilis*) in adults remain high, with nearly one million new infections each day [1, 2]. The spread of STIs leads to many sequelae and complications that disproportionately affect women and adolescents [3–5]. WHO implemented the first (2006–2015) and the second (2016–2021) strategy on STIs with the goal of ending STIs epidemics as a public health concerns by 2030 [6, 7]. In general, low- and middle-income countries (LMICs) have higher estimated burdens of STIs than high-income countries (HICs) [2]. In these countries, STIs diagnostic tests are largely unavailable, expensive and technically demanding on microbiological surveillance systems, so that, surveillance of clinical syndromes called.

syndromic approach is easier to establish in public health. Although this approach according to the WHO guidelines obtains the highest marks in terms of development rigor with ongoing training every 2 years and is the surveillance system that can best represent the epidemiology of STIs in general population, they may result in an underestimated burden due to asymptomatic cases and have moderate specificity and sensitivity [8, 9].

In the Maghreb (region of North Africa bordering the Mediterranean Sea), some studies have focused on sex workers and women requesting abortion whose STI diagnostic criteria were based on laboratory results (endocervical, urine and vaginal sample) [10–13]. However, few studies have used clinical surveillance data to establish the trend and incidence of certain curable STIs in the general population [14, 15].

Quantifying the incidence of STIs is important for planning and promoting sexual health interventions. Thus, it is necessary to have current data to evaluate the situation in the Maghreb and more specifically in Tunisia to confirm the hypothesis of the decreasing number of STIs and to possibly reach the desired objective by WHO for 2030.

The aim of this study was to determine the incidence of symptomatic STIs among women during eleven-years in the governorate of Monastir and to evaluate the effectiveness of syndromic management approach to reach the 2030 WHO goals.

## Methods

We conducted a descriptive study including all female episodes of STIs from January 1, 2007 to December 31, 2017.

Monastir Governorate is one of the twenty-four governorates of Tunisia. It is situated in northeastern Tunisia. It covers an area of 1019 km^2^ (393 mi^2^) and is divided into thirteen delegations*.* In 2014, the population of Monastir represented 4.99% of the Tunisian population [16]. In 2015, the total number of Basic health centers (BHC) in the governorate of Monastir was 101 [17]. Primary care sectorization is regulated to provide treatment to people in their own region. Each BHC only receives patients who are geographically assigned to it. Therefore, each patient can only consult in one BHC except urgent cases.

Participants: From January 1, 2007 to December 31, 2017, we included all new episode of female STIs diagnosed and treated according to the WHO syndromic approach summarized in flowcharts (clinical algorithm) [18] (Appendix 1). A person with repeated infections may count more than once. The components of case management included: taking history, examination, correct diagnosis, early and effective treatment, advice on sexual behaviour, promotion and/or provision of condoms. All STIs cases diagnosed by the syndromic approach were registered by the physicians or midwifes of each BHC prospectively in a form that was reported to the regional direction of primary health at the end of each month and recorded on “epi-info” software.

All women in all age groups who consulted the midwives or physicians of each BHC in Monastir governorate for sexual health services and diagnosed by the syndromic approach from January 1, 2007 to December 31, 2017 were included in the study. Other STIs cases, which were not diagnosed by the syndromic approach such as vesicular lesions (herpes), chancroid and genital warts, were not included in this study. Patients who lived in other areas were excluded from the study.

Data included variables related to age, sex, consultation date and diagnosis.

The syndromic approach included four STIs presented clinically by four symptoms: Pelvic Pain (PP), Vaginal Discharge (VD), Genital Ulceration (GU) and Ureteral Discharge (UD).VD include cervicitis, vaginitis and vaginosis whose symptoms are respectively discharge of muco-pus through the cervix, smelly fluid vaginal discharge and non-specific vaginal discharge (thicker, greenish, smelly) [18]. For each syndrome, there are well-defined infectious agents incriminated in its symptomatology [18] (Table 1).

**Table 1:**
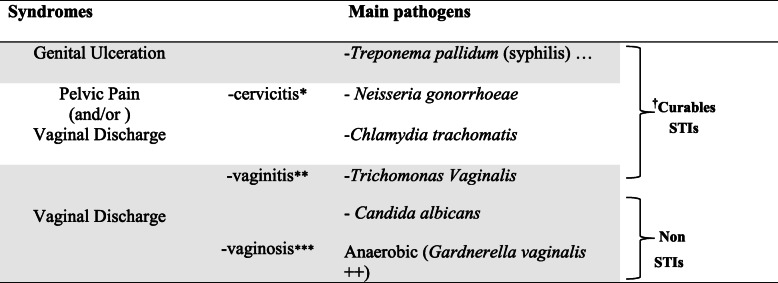
Pathogens according to each symptom in women according to WHO

* Discharge of muco-pus through the cervix

** Smelly fluid vaginal discharge

*** Non-specific vaginal discharge (thicker, greenish, smelly)

*STIs* Sexually transmitted infections, *Non STIs* Non Sexually transmetted infections

^**†**^**: WHO** defined *chlamydia, gonorrhoea, trichomoniasis* and syphilis as curable STIs

As data collection from the registry of STI surveillance was conducted retrospectively, data available on personal and contact information was not exhaustive and there could be a risk of under reporting.

Tunisian National Institute of Statistics provides each year the estimation of female population and their distribution by age and governorate [19].

The crude incidence rate (CIR) was calculated by dividing the total number of episodes from 2007 to 2017 in Monastir by eleven, and by the female population in Monastir then multiplied by 100,000. It was expressed as the number per 100,000 person-years (/100,000 PY). CIR= $\left(\frac{\left(\mathrm{total}\ \mathrm{number}\ \mathrm{of}\ \mathrm{episodes}\right)}{11}\right)/\left(\mathrm{the}\ \mathrm{average}\ \mathrm{female}\ \mathrm{population}\right)\Big)\ast 100000$. The average population was calculated as follows: ((the sum of estimated female population each year) /11) which was 263,567.

The annual CIR was calculated as follows $\left(\frac{\left(\mathrm{total}\ \mathrm{number}\ \mathrm{of}\ \mathrm{episodes}\ \mathrm{each}\ \mathrm{year}\right)}{\mathrm{the}\ \mathrm{female}\ \mathrm{population}\ \mathrm{each}\ \mathrm{year}}\right)\ast 100000.$

The age-standardized incidence rate (ASR)/100,000 PY was calculated using the world standard population according to the WHO statement of 2013 [20].

The Confidence Intervals (CIs) for CIR and ASR were calculated at a confidence level of 95%.

Data were verified and analyzed using IBM SPSS Statistics version 22.0 software (IBM Corp., Armonk, NY, USA). Categorical variables (age group) were recorded as numbers and percentages. Chi-square test was used to study the association between STIs syndrome and age groups. Linear regression was used to estimate the trends in notified disease according to sex and age group. A *p*-value of < 0.05 was considered statistically significant.

### Ethical considerations

The study was conducted under Good Clinical Practice conditions and according to ethical standards collections. Data collection and analysis were labelled accordingly to maintain anonymity. The ethics committee of Faculty of medicine of Monastir approved the protocol.

## Results

During 11 years, 40,388 episodes of four female curable STIs were diagnosed by syndromic approach in BHC in Monastir governorate with a mean of 3671 episodes /year. Overall, the crude incidence rate (CIR) was 1393 (95%CI; 1348–1438) / 100,000 PY being the highest (3453/100,000 PY) in the 30–39 age group. ASR was 1328 (95% Confidence Interval (CI); 1284–1372) /100,000 PY. VD was the most common syndrome (a CIR of 1170/100,000 PY) and specifically vaginosis with a CIR of 907/100,000 PY (Table 2).

**Table 2:** Crude and age standardized prevalence rates of woman, by age groups and syndromes of sexual transmitted infections diagnosed by syndromic approach in Monastir (2007–2017)

	Cases (%) (11 years)	CIR (95%CI) ^**a**^	ASR (95%CI) ^**a**^
**Overall**	40,388 (100)	1393 (1348–1438)	1328 (1284–1372)
**Age groups (Years)**			
0–4	6 (0.0)	2.24 (−3–8)	
5–14	77 (0.2)	15 (4–27)	
15–19	1060 (2.6)	407 (326–488)	
20–29	9986 (24.7)	1630 (1524–1735)	
30–39	15,318 (37.9)	3453 (3274–3631)	
40–49	10,268 (25.4)	2886 (2703–3049)	
≥ 50	3673 (9.1)	700 (625–774)	
**Syndromes**			
PP	6126 (15.2)	211 (193–228)	
GU	336 (0.8)	11 (7–15)	
VD	33,926 (84.0)	1170 (1129–1211)	
Cervicitis	1099 (2.7)	37 (29–45)	
Vaginitis	6519 (16.1)	224 (206–242)	
Vaginosis	26,308 (65.1)	907 (871–943)	

^a^per 100,000 person year, *CIR* crude Incidence rate, *ASR* age standardized rate, *95%CI* 95% Confidence Interval, *PP* Pelvic Pain, *VD* Vaginal Discharge, *GU* Genital Ulceration

**Fig. 1:**
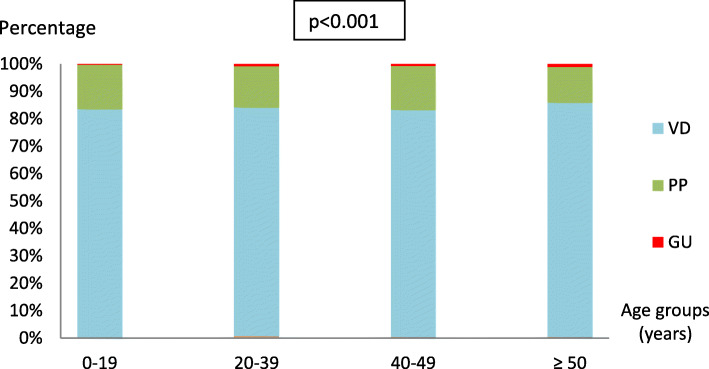
Distribution of syndromes according to age groups Monastir (2007–2017). PP: Pelvic Pain; VD: Vaginal Discharge; GU: Genital Ulceration

In all age groups the VD was the most frequent syndrome (*p* < 0.001) (Fig. 1). For all syndromes, the age group most affected ranged from 20 to 39 (*p <* 0.001) (Table 3).

**Table 3:** Distribution of STIs syndrome by age groups in Monastir (2007–2017)

AGE GROUPS	0–19 n (%)	20–39 n (%)	40–49 n (%)	≥ 50 n (%)	Overall n (%)	P*
Syndromes						
PP	186 (3.0)	3818 (62.8)	1642 (26.8)	480 (7.8)	6126 (100)	< 0.001
GU	3 (0.9)	213 (63.4)	80 (23.8)	40 (11.9)	336 (100)	0.038
VD	954 (2.8)	21,273 (62.7)	8546 (25.2)	3153 (9.3)	33,926 (100)	< 0.001
Cervicitis	31 (2.8)	817 (74.3)	214 (19.5)	37 (3.4)	1099 (100)	< 0.001
Vaginitis	132 (2.0)	4467 (68.5)	1491 (22.9)	429 (6.6)	6519 (100)	< 0.001
Vaginosis	791 (3.0)	15,989 (60.8)	6841 (26)	2687 (10.2)	26,308 (100)	< 0.001

*PP* Pelvic Pain, *VD* Vaginal Discharge, *GU* Genital Ulceration, *Chi2-square test

**Fig. 2:**
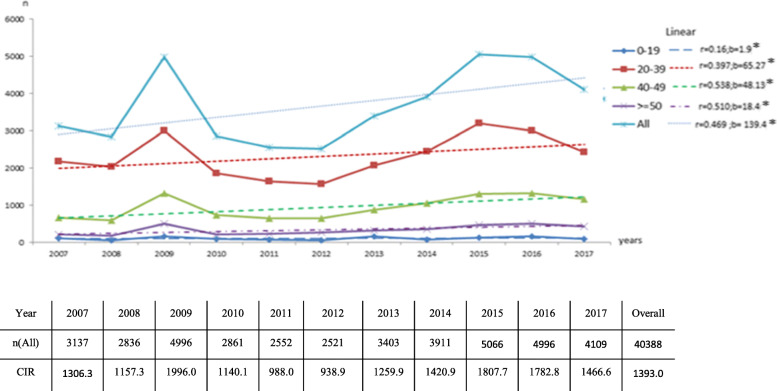
Trends of STI’syndromes by age groups (2007–2017) in Monastir

The distribution of syndromes by years showed that the highest rate was observed in 2009 with a number of 4996 (CIR =1996/ 100,000 PY). The lowest rate was recorded in 2012 with 2521 episode (939/ 100,000 PY). All syndromes increased over time (b = 139.396; *r =* 0.469; *P* < 0.001). All age groups had a positive trend. The age groups with the highest growth rate were those aged 20 to 39 and 40 to 49 in 11 years with standardized coefficient of 65.27 (*r =* 0.39;*p <* 0.001) and 48.13 (*r =* 0.538;*p <* 0.001) respectively (Fig. 2).

**Fig. 3:**
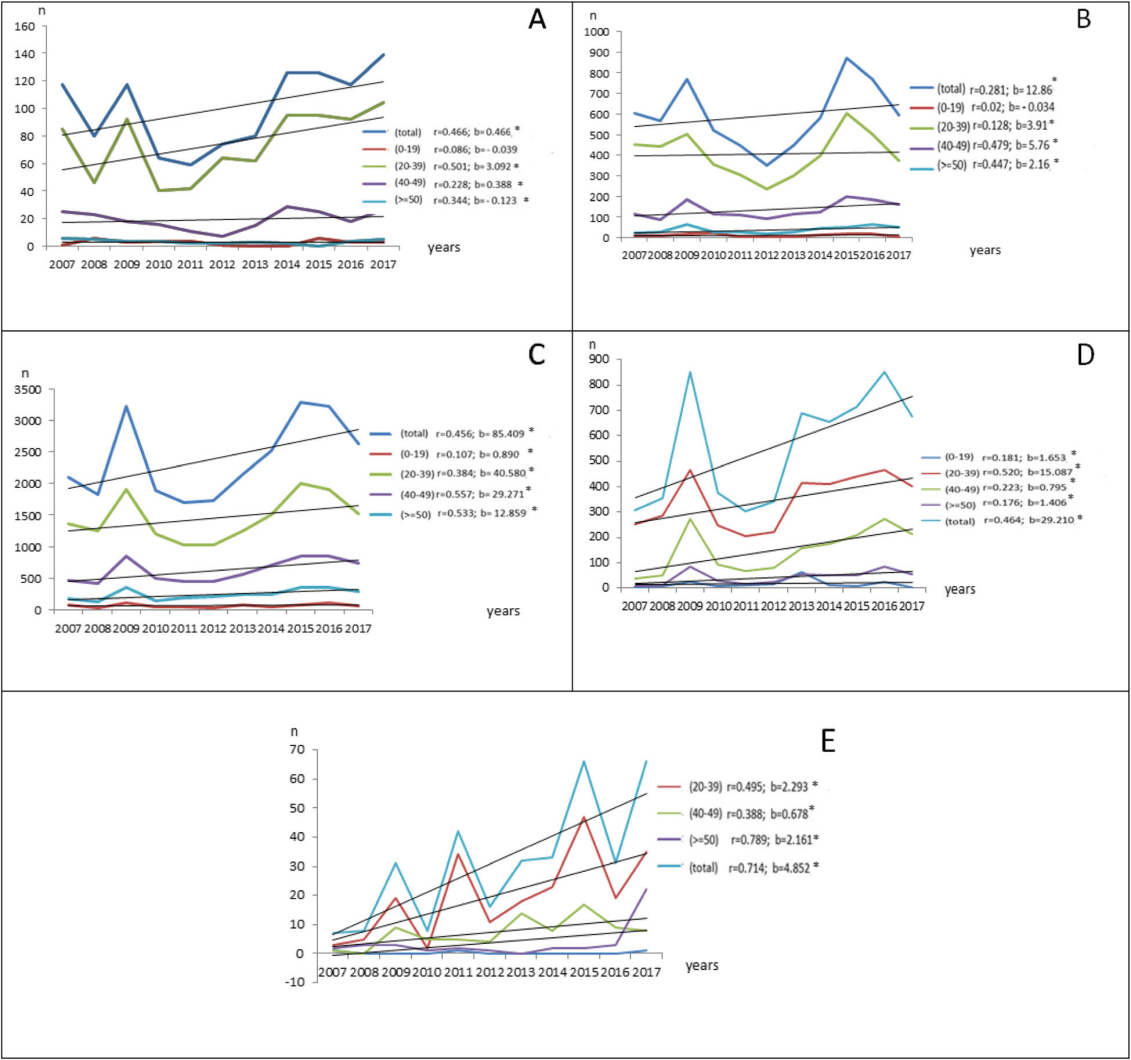
Distribution of each STI’syndromes by years and age groups (2007–2017) in Monastir. **A: cervicitis:** for age group of 20–39 years a positive trend(*r =* 0.501; b = 3.092; *p <* 0.001) was established. **B: vaginitis**: for age group of 20–39 years a positive trend (*r =* 0.128; b = 3.91; *p* < 0.001) was established. **C: vaginosis**: for age group of 20–39 years a positive trend(*r =* 0.384; b = 40.580; *p <* 0.001) was established. **D: pelvic pain:** for age group of 20–39 years a positive trend (*r =* 0.520; b = 15.087; *p <* 0.001) was established. **E: Genital ulceration:** for age group of 20–39 years a positive trend *r =* 0.495; b = 2.293; *p <* 0.001 was established. *p < 0.001

Episodes of cervicitis, vaginitis, vaginosis, PP and GU increased over time specifically for age group of 20–39 years (Fig. 3).

## Discussion

The incidence rate of STIs episodes among women diagnosed with the syndromic approach was high. Cases increased over time for all age groups and symptoms. VD and especially vaginosis was the most common recorded syndrome. For all syndromes, women aged 20 to 39 were the most affected age group.

Globally, 2012 and 2016 estimates of the incidence of the four curable STIs (*chlamydia, gonorrhea, trichomoniasis* and syphilis) remain high among women of reproductive age [1, 2, 21].

World estimates have shown that among the pathogens that cause these four diseases, *Trichomonas Vaginalis* is the most frequent in women 5.3% (95% uncertainty interval UI:4.0–7.2) followed by *C. trachomatis* 3.8% (95%, UI: 3.3–4.5); *Neisseria gonorrhoeae* 0.9% (95% UI: 0.7–1.1) and *Treponema pallidum* 0.5% (95% UI: 0.4–0.6). The same order of pathogens according to their involvement in STIs was also recorded in the East Mediterranean Region (EMR) with a predominance of *Trichomonas Vaginalis: 4.7*% (95% UI: 3.3–6.7) followed by *C. trachomatis* 3.8% (95% UI: 2.6–5.4); gonorrhea 0.7% (95% UI: 0.5–1.1) and syphilis 0.7% (95% UI: 0.4–1.0) [1, 2, 22]. This frequency order of syndromes was the same as our findings.

The lower rate of GU (syphilis) episodes than in other areas in the East Mediterranean Region may be explained by the fact that in Tunisia, as part of the national perinatal program, screening for syphilis infection is compulsory for the prenuptial examination for both couples and likewise compulsory for pregnant women during the first prenatal visit. This may also explain that the prevalence of infection with syphilis is lower than other pathogens.

In our results,when excluding vaginosis which are in most cases caused by a non sexually transmetted pathogens “*Gardnerella vaginalis”* [23], we found that vaginitis (mainly caused by *Trichomonas Vaginalis*) was the most frequent syndrome followed by pelvic pain and cervicitis (mainly caused by *C. trachomatis* and *Neisseria gonorrhea*) .

In Tunisia, several studies on the epidemiological characteristics of sexually transmitted infections in women found a high prevalence of VD and the pathogen *C. trachomatis* in women of reproductive age [8, 12]. In Morocco, a 1995–2015 study of reported cases of VD showed that the prevalence of *C. trachomatis* was 3.8%; IC95%[2.1–6.4] and that of *Neisseria gonorrhoeae* was 0.37% IC95%[0.14–1] [15]. A 2019 meta-analysis found that *chlamydia trachomatis* infection had the highest mean prevalence (80.3% (95% CI = 53.2–97.6%)) among female sex workers in the Middle East and North Africa (MENA) [11] .

In our study, nearly two-thirds of the genital symptoms of STIs were vaginosis (65.1%) with the highest estimated CIR (907/100,000 PY), compared to other genital symptoms of STIs. A meta-analysis published in 2019 showed that the overall prevalence of bacterial vaginosis in the global population was high, ranging from 23 to 29% in all regions (Middle East and North Africa, 25%; sub-Saharan Africa, 25%) [24, 25]. This variations in bacterial vaginosis between countries and between ethnic groups within countries are well described [26].

Consistent with our findings, WHO prevalence estimates of the four curable STIs among women have shown an overall positive trend from 2012 to 2016 globally and especially in the EMR [22]. The incidence of STIs has been increasing in Monastir in all age groups. This could be explained by the fact that the beliefs of the population have become more liberal and that sexuality has become a little less taboo, so that the use of care has become less discriminatory over time. On the other hand,the syndromic approach does not allow vaginal sampling, hence the possibility of misdiagnosis, inappropriate prescribing which can also lead to recurrence of episodes of infection and long-term microbial resistance.

In our study, most STI cases were in the 20–39 age group. In fact, it is the sexually active group at high risk of being more susceptible to behavioral exposure to STIs [27–29] and it is the age of reproduction where the search for symptoms of STI infection may be more sought during prenatal visits and pregnancies.

Our study had some limitations. STIs Surveillance at primary health care level was based on syndromic data and not on biological tests. Since each symptom has multiple potential agents, this study based on syndromic data cannot provide accurate statistics on the frequency of STI pathogens. Indeed, according to studies carried out in Tunisia and a prospective observational cohort study in South Africa [3, 8], the diagnosis according to the syndromic approach had low rates of sensitivity and specificity, which suggests an underestimation of the actual rate of STIs in our study due to the fact that asymptomatic cases can only be diagnosed by appropriate laboratory tests.

On the other hand, many cases of VD are not caused by sexually transmitted infections such as cervical ectopy, foreign bodies, retained tampon, vulval dermatitis, non-sexually transmitted infection like some bacterial vaginosis and candida infections [30, 31].

Following these results, we recommend several actions. First, improve the knowledge and awareness of people regarding STIs since early age through focusing sexual education efforts in secondary schools. Second, consider periodic routine microbiological etiology surveillance to guide syndromic management of STIs and minimize misdiagnosis. Third, given that STIs and HIV are synergistic [32, 33] and that HIV testing in Tunisia is carried out in a few specific basic care centres and that testing is not systematic for all STI consultants(It is an anonymous test and its acceptance by the community remains limited), we suggest to properly screen for contacts and increase the rigour of HIV testing by systematically referring STI cases to the Centre’s anonymous and free HIV screening.

## Conclusion

The incidence rate of STIs among women diagnosed with the syndromic approach was high, consistent with the global evidence. It showed a positive trend over 11 years for all age groups and syndromes. VD was the most common recorded syndrome. For all syndromes, women aged 20 to 39 were the most affected age group. To achieve the World Health Organization goal of ending STIs epidemics by 2030, several measures should be reinforced in low and middle-income countries such as strengthening the STI surveillance system, additional preventive interventions, prompt identification, correct treatment, and partner tracing to stop the spread of these infections.

## Supplementary Information

**Additional file 1: Appendix 1**. The WHO syndromic approach summarized in flowcharts (clinical algorithm)

## Data Availability

The data that support the findings of this study are available from the regional direction of primary health of Monastir but restrictions apply to the availability of these data, which were used under license for the current study, and so are not publicly available. Data are however available from the authors upon reasonable request and with permission of the regional direction of primary health of Monastir.

## Abbreviations

ASR: Age-Standardized Incidence Rate
BHC: Basic Health Centers
CIR: Crude Incidence Rate
EMR: East Mediterranean Region
GU: Genital Ulceration
HICs: High-Income Countries
HIV: Human Immunodeficiency Virus
LMICs: Low- and Middle-Income Countries
MENA: Middle East and North Africa
PP: Pelvic Pain
STIs: Sexually Transmitted Infections
VD: Vaginal Discharge
WHO: World Health Organization
