# Supplementary figures and images for: Syndromic surveillance of female sexually transmitted infections in primary care: a descriptive study in Monastir, Tunisia, 2007─2017
Source: BMC Public Health. 2021 Sep 6;21:1625. doi: 10.1186/s12889-021-11647-2 (PMC8420027; doi:10.1186/s12889-021-11647-2)

| 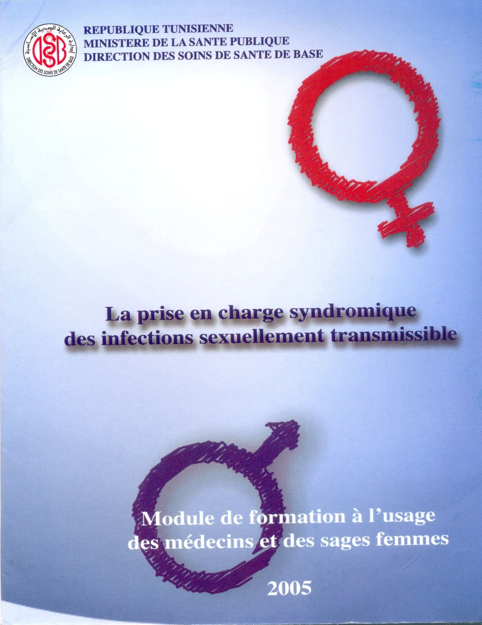 | |
| --- | --- |
| 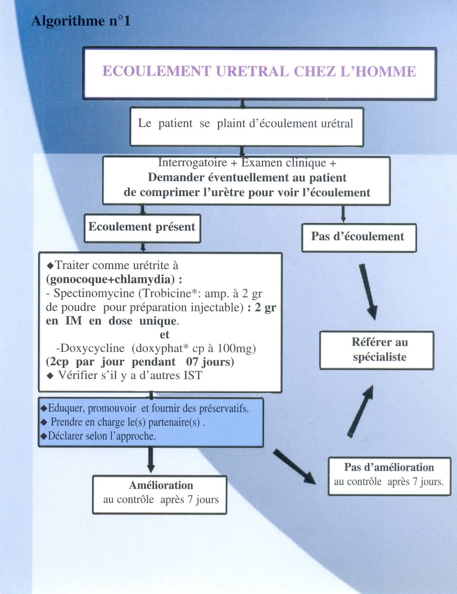 | 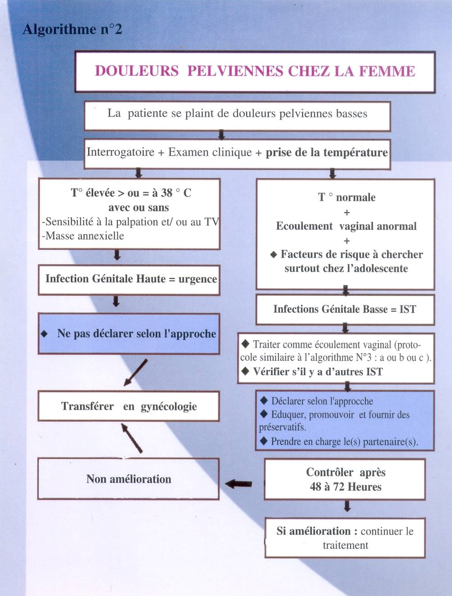 |
| 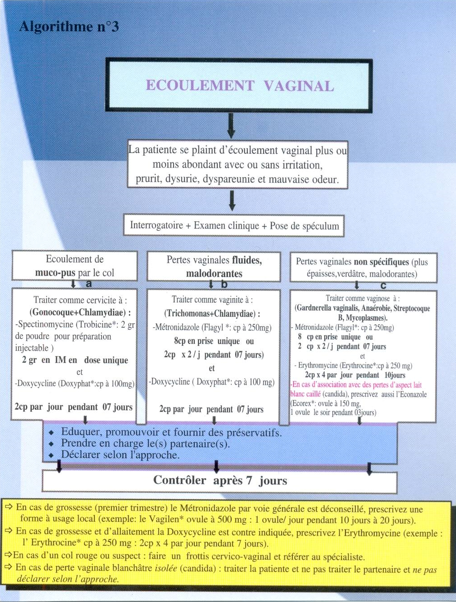 | 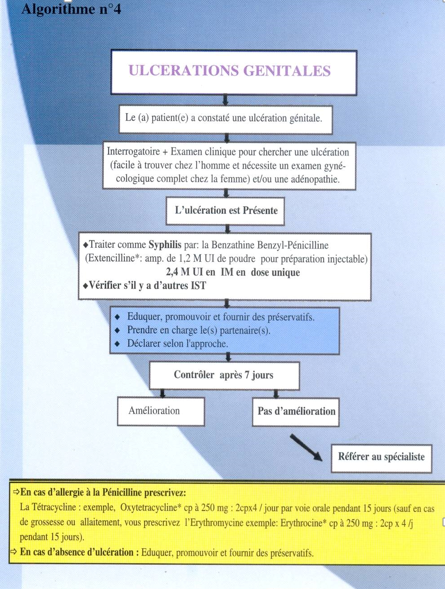 |

Appendix 1:The WHO syndromic approach summarized in flowcharts (clinical algorithm)

Supplement: Supplementary file 1 — Additional file 1: Appendix 1. The WHO syndromic approach summarized in flowcharts (clinical algorithm) [file 12889_2021_11647_MOESM1_ESM.docx]
